# Healthcare-seeking experiences of older citizens in Bangladesh: A qualitative study

**DOI:** 10.1371/journal.pgph.0001185

**Published:** 2023-02-08

**Authors:** Abdur Razzaque Sarker, Irfat Zabeen, Moriam Khanam, Ruckshana Akter, Nausad Ali

**Affiliations:** 1 Population Studies Division, Bangladesh Institute of Development Studies (BIDS), Dhaka, Bangladesh; 2 Institute of Applied Health Research, University of Birmingham, Birmingham, United Kingdom; 3 Institute of Health Economics, University of Dhaka, Dhaka, Bangladesh; 4 Ministry of Education, Dhaka, Bangladesh; Kamuzu University of Health Sciences (KUHes), MALAWI

## Abstract

Despite improvements in many health indicators in the last few decades, providing access to affordable and quality healthcare for older citizen remains a considerable challenge in Bangladesh. This study aimed to understand individuals ‘experiences regarding their healthcare-seeking, treatment cost, accessibility and coping mechanisms for the promotion of appropriate strategies to enhance the quality of life of the older citizens of Bangladesh.A qualitative descriptive approach was used in this study. A total of 27 In-Depth Interviews (IDIs) were conducted in a district in Bangladesh with older people between January and February 2020, where gender distribution was equal. Face-to-face interviews were conducted by trained and experienced interviewers regarding healthcare-seeking and accessibility, affordability, and healthcare coping strategy. Thematic analysis was conducted to analyse the data. It was found that the health condition of the older population is not satisfactory. Most of them had been suffering from several diseases such as benign tumor, chronic kidney disease, body aches, gastric ulcers for a longer period of time. The majority of the participants were suffering from multiple non-communicable diseases while diabetes and hypertension were the foremost of all diseases. This study provides insight into the challenges of managing healthcare services for older citizens in Bangladesh. Healthcare facilities were available, but high out-of-pocket payments, lack of caregivers, and time distance created a barrier to the service provision. The findings indicated that geriatric care policymakers and service providers should prioritize the older-friendly health infrastructures with affordable cost of treatment for the betterment of the health status of older citizens in Bangladesh.

## Introduction

Despite improvements in many health indicators in the last few decades, providing access to affordable healthcare remains a considerable challenge worldwide, especially for older people living in low- and middle-income countries (LMICs) like Bangladesh [1–3]. In recent years, many countries have experienced significant changes in the age composition of their population, particularly, the population aged 60 years and more has steadily increased in numbers [4]. Changing dynamics in birth and death rates, declining fertility rates, and increasing life expectancy lead to these changes in the global age structure [5]. The demographic structure is changing rapidly in Bangladesh. According to the national estimation conducted by the Bangladesh Bureau of Statistics, approximately 8% of the total population is 60 years or above, which is projected to be increased to 11.5% (21.526 million) by 2030 in Bangladesh [6,7]. The overall life expectancy of the population is also increasing (67.7 years in 2010 and 72.32 years in 2020) which creates both opportunities and public health challenges for Bangladesh [8]. It is expected that everyone should hold better health to live a longer life which will enable them to provide valuable economic, social as well as cultural contributions. Due to shifting disease patterns from communicable diseases (CDs) to non-communicable diseases (NCDs), various non-communicable health issues such as, sensory and cognitive impairments, dementia, physical inactivities, and even the risk of falling during daily activities have become more prevalent among the older population which requires regular health services from various healthcare facilities [9,10]. Further, the gradual rising of nuclear families makes older people more isolated and pushes them to be vulnerable with respect to physical and mental health in Bangladesh [11]. Thus, older citizens with a high risk of diseases and disabilities would put urgent demands on the health systems while Bangladesh is not well prepared to meet such healthcare demands. Documenting the current experiences of older people in seeking health care can inform policy decisions on improving the health system’s performance in tackling the growing health care needs of the elderly.

This study tried to document the health-related problem and care-seeking patterns of the older citizen. To understand the care-seeking pattern we followed the theoretical framework proposed by Kasl and Cobb using three important behavior such as- health behavior, illness behavior, and sick role behavior [12,13]. Kasl and Cobb discussed that the likelihood of such behavior is a function of the perceived amount of threat and the value of the behavior. The amount of threat depends on health matters, susceptibility to the disease, and the consequences of diseases while the value of the behavior often depends on the perceived probability of the action that will prevent the disease and the cost of taking the action [12,14]. Indeed, the pattern of diseases and care-seeking behavior of older citizens is heterogeneous and often more complex than that of other ages and thus requires more attention. Many older citizens are often unable to pay for these services and even out of reach of healthcare services [15–17]. Further, aging causes functional deterioration and vulnerability which triggers to increase in the household’s healthcare expenditure [18]. Albeit, the government, and related authorities are often concerned that older citizens will inevitably increase public health care expenditures in near future, public investment is still focused on maternal and child health issues in Bangladesh [19].

The life expectancy of people is increasing worldwide, and the pace of population aging is much faster than in the past. Bangladesh is also facing such challenges and would put urgent demands on the health system. Although a body of literature in Bangladesh focuses on healthcare utilization and associated costs among various population groups, there is little evidence of a study targeting older citizens regarding their views and experiences about healthcare services in Bangladeshi context [8,19–21]. To address this public health concern, this research aimed to understand individuals’ experiences of older people in Bangladesh regarding healthcare-seeking and accessibility, affordability, and coping strategy. The current study also considers the social and cultural aspects of healthcare utilization during their sickness. As such, the present study attempted to bridge the knowledge gap and shed light on emphasizing the promotion of the healthcare condition of senior citizens. The older population is now considered an important contributor to age structure; therefore, without securing their healthcare, universal health coverage (UHC) cannot be achieved.

## Methods

### Study design

A qualitative descriptive approach was used to understand the experiences of older citizens in terms of their health condition and health care needs, accessibilities, treatment costs, and related factors [22–25]. The present study employed in-depth interviews (IDIs) that provide a comprehensive summary of events reported directly by older adult individuals. This approach delivers more in-depth and circumstantial evidence while providing a comprehensive summary of events in the usual language of the participants [26]. A total of 27 IDIs were conducted up to data saturation with no refusal.

### Study settings & population

This study was conducted in the Tangail district of Bangladesh, where the first ever government-initiated health protection scheme was piloted. Tangail is also nearby to the capital city of Bangladesh where various types of healthcare facilities including tertiarly and specialized healthcare are available. Further, due to minimum-distance and better communications, people can avail the healthcare services from the capital city of Bangladesh. Older citizens or the elderly population are defined as people of sixty years or more, according to the National Policy on Elderly People in Bangladesh [27]. The older citizens residing in this district for a minimum of 1 year were eligible as respondents. For each in-depth interview, one after every 3^rd^ household was targeted and then if any older citizens were found on that very household, were selected for the interview. Additional probing that provided insights relevant to our study was also used. Repetitions within the data were noted, suggesting that the data reached saturation [28]. After interviewing these participants, the research team were satisfied that thematic saturation was reached and recruitment ceased. Therefore, a total of 27 In-Depth Interviews (IDIs) were conducted where gender distribution was equal. Face-to-face interviews were conducted by trained and experienced interviewers using specific interview guidelines.

### Data collection

Considering the convenience and availability of the participants, face-to-face individual in-depth interviews were conducted between January and February 2020. The interviews lasted between 40–60 minutes and were all conducted in Bangla- the country’s local language. The study participants were contacted via in-person visits in their households, and the objectives were described clearly. The household list was collected from the local government of Bangladesh. An interview date was fixed for them, who agreed to participate in the in-depth interview. The interviewers were well trained on the guidelines and had significant experience using qualitative data collection tools. This in-depth guideline was adopted from published qualitative literature based on healthcare utilization and barriers and then finalised considering both cultural and socio-demographic aspects of the Bangladeshi context [29]. Participants were first asked to provide written informed consent to participate, and then verbal consent was recorded at the beginning of the interviews. Each discussion between the participant and interviewer was recorded using an audio recorder with the respondents’ permission. Initially, the questions were broad, according to the guidelines, and later probing was applied to achieve more detailed responses.

### Data analysis

A content analysis was conducted to analyse the data. To ensure reliability in analyzing the interviews, researchers independently checked all the interviews. Transcriptions were made from each of the recorded interviews by the interviewers in their native language (Bangla), and the transcripts were read with field notes for an overall understanding. All transcripts were translated into English by a bilingual translator directly involved in the study. Later, the transcriptions and translations were checked twice by other investigators. Next, the transcriptions were structured using the framework method proposed by Gale et al. and categorized the answers into five themes [30]. Thematic analysis was conducted to ensure methodological accuracy and transparency of qualitative data analysis. To carry out a thematic analysis, several phases were followed according to the methodology adopted by Braun and Clarke, which included familiarizing the data, generating initial codes, defining and reviewing themes, and drawing up general interpretations [31]. Data were manually coded according to meaningful statements (issues, highlights, concerns, and accomplishments) about older health-related experiences and then categorized by the team investigators. Contents were compared across codes, and key concepts were recognized where core themes were identified. A qualitative analysis expert cross-checked the themes for common agreement and refined the identified themes. The investigators finally listed specific themes based on the guidelines (S1 Text) and code categories that included participants’ perceptions, personal beliefs, and understanding. Finally, an evaluation of the themes with a re-reading of the interviews was performed to ensure that the participants’ insistence, meaning, and perception were precisely captured. All explications provided by the respondents were categorized into five distinct themes that described the experiences and expectations of older citizens about healthcare accessibilities and the future scope of improvement.

### Ethics statement

This study was performed in line with the principles of the Declaration of Helsinki. The research was approved by the institutional review board at Bangladesh Institute of Development Studies, IRB Protocol#: PSD/01/2019.20/03/REF/HSBAECB. Participants were informed in advance that their participation was voluntary and that all information provided would remain confidential. All study participants provided written consent prior to data collection.

## Results

A total of 27 interviews were conducted up to data saturation with no refusal. Table 1 presents the basic demographic characteristics of the participants. The majority of the respondents were aged between 60–65 years (59%), and most of them (56%) had no formal education. 18% of the total respondents had a monthly household income of 10,000−15,000 BDT, while 48% were earning less than 10,000 BDT.

**Table 1:** Descriptive characteristics of the respondents.

Characteristics	Frequency	Percentage
Gender		
Female	13	48.40
Male	14	51.85
Age in Years		
60–65	16	59.26
66–69	5	18.52
≥70	6	22.22
Education		
No formal education	15	55.56
Up to primary	7	25.93
Up to secondary	4	14.81
Higher secondary	1	3.70
Monthly Household Income		
Less than 10,000	13	48.15
10,000–15,000	5	18.52
15,001–20,000	2	7.41
More than 20,000	5	18.52
Don’t Know	2	7.41
Total	27	100

All elucidations provided by the participants were identified under five themes: health condition, care-seeking pattern, expectations, treatment cost and out-of-pocket cost issues and coping strategiesThese themes are presented using the participants’ direct voice to explore the in-depth context and detailed meaning of specific themes that optimally described their experiences. Theme description is quoted in italics (Table 2).

**Table 2:** Themes emerged from the data.

Theme	Sub-Theme
1. Assessment of health status	1.1 Current Health Status
	• “Almost all the respondents’ health condition were poor”
	1.2 Communicable vs. Non-Communicable Diseases
	• “Communicable diseases are mostly common for each respondent”
	1.3 Long-term illness
	• “Carrying illness till death”
2. Healthcare seeking pattern	2.1 Decision of choosing health facility
	• “Prone to seek health care from private clinic/hospital”
	2.2 Preferable option for healthcare
	• “District public hospitals are preferable than sub-district level health facilities”
3. Expectation from service provider	3.1 Quality improvement
	• “Quality health care is first priority”
	3.2 Affordability and accessibility
	• “Affordability and Transportation convenience play crucial role for seeking care”
	3.3 Other issues
	“Quality of service provider and environment is also important in deciding care seeking”
4. Out of pocket cost is one of the barrier in accessing healthcare	4.1 Treatment cost
	• “Consultation fees should be reduced for the poor older people”
	• “Surgery and operation costs are not affordable”
5. Coping strategies for the health care expenditure	5.1 Consequences
	• “Managing health expenses is a unbearable burden for the older”
	5.2 Suggestions from the respondents
	• “Multiple suggestions are carried out to mitigate healthcare expenditure”

### Theme 1: Health condition of older people

Participants recounted their experiences during sickness, describing diverse individual illnesses and varied patterns of disease symptoms presenting throughout the body. Almost all of the respondents mentioned that their health condition was abysmal. The majority of the respondents reported that they had been suffering from several diseases such as a benign tumor, chronic kidney disease, body aches, and gastric ulcer for a long time. Some of them stated that they could not even walk or participate in regular work. Most participants suffered from multiple non-communicable diseases (NCDs), while diabetes and hypertension were the foremost of all diseases. The health condition of older people was so fragile that some of them were not in ambulatory condition. Eye infections, benign tumors, chronic kidney disease, body aches, and gastric ulcers were some major causes of illness. One-third of them had been suffering from those diseases for over 10 years. One male respondent aged (64) stated, “*My health condition is declining as my heart is weak*, *the bulb is damaged and there is a hole in my rectum*. *Though I had an operation 35 years ago*, *there is no sign of improvement yet*.” Most of the respondents explained their miserable health condition by stating that they had been suffering from such a devastating illness for a long time. Indeed, some came to know about their diseases after a major clinical operation (e.g. tumor, eye diseases, and diabetes) and even after experiencing a stroke. Consequently, they are now living with continuous medication which is often unaffordable and are worried about future medication in this regard. One older respondent (68) expressed, “*I have high blood pressure*, *gastric ulcers*, *and pains in a different organ*. *A long time ago*, *maybe 2 years or more*, *I had a stroke but I had no sense at that time*. *Since then*, *I have been on medication and I have to carry on till death*.” The other respondents (69) stated, “*Many of my friends and relatives are suffering from hypertension and diabetes and some of them advised me to get examined by a physician for diabetes and hypertension*, *and then the doctors confirmed me about these diseases*.*” It was almost eight (8) years ago and I am still suffering*.*”*

With ageing, people are more likely to experience multiple health conditions which leads them to seek healthcare services which also differ in several aspects. Thus, our second theme features the pattern of care-seeking behaviour of this group.

### Theme 2: Healthcare-seeking pattern

Healthcare-seeking pattern refers to the sequence of remedial actions that individuals undertake to rectify perceived ill health which is essential to provide need-based healthcare delivery, particularly, for the older population, and to make the healthcare system more pro-poor. Almost every respondent was affirmative about the private clinic or hospital when they had been asked about the source of healthcare. Indeed, pharmacies were their first choice due to the convenient and easily approachable nature. A woman (65) replied, *“I take my medicines regularly from pharmacies because my doctor advised me to take my medicines regularly*, *and most of the pharmacies are near to my door*.*”* The other respondent (70) said that *“the drug seller is very well known to me and I can trust easily*.*”* Older people prioritized private clinics/hospitals as they believed quality services are available there than any other institutions. Some of them were used to going to the private facilities because their relatives or close persons worked there by which they could avail quality services at a low-cost (at least without doctor fees). One of the older citizens stated (65), “*a doctor from Mymensingh Sadar consults with patients in Life Care Hospital (a private hospital)*. *He was one of my relatives and I used to go to him for care*. *Three months ago*, *I have consulted with him and he gave me certain medications including antibiotics*. *He also suggested about going to another doctor of the same private institution for further treatment*.” In most of the cases, the private healthcare facilities were either adjacent to their houses or at the center of their city where they can easily go with existing transportation. One of the respondents (67) replied, “*Lack of older-friendly transportation is a big problem for me*, *as I cannot sit properly with the available transportation and also I have no extra family member who could help me as caregiver*.”

Though the majority of the study participants preferred private clinics, they often visited district public hospitals for regular checkups for chronic diseases like diabetes, asthma, heart diseases, eye operation, and kidney operation as public hospitals are highly subsidized in Bangladesh. Indeed, we observed that the participants choose district public hospitals when it comes to an emergency situation. Very few numbers of them went to Dhaka, the capital of Bangladesh for consulting specialized doctors and for better care. On the other hand, the participants were often unwilling to visit sub-district level public hospitals due to the lack of medical personnel and medical technologies. Nevertheless, very few of them visited these places and had to buy medicines from the pharmacy or other sources due to the scarcity of medicines in those facilities. A statement from a respondent (70), “*at first I went to Modhupur Hospital (sub-district level hospital) for treating asthma-related problems*, *but that wasn’t solved*. *That’s why*, *I visited Mymensingh Sadar Hospital (a district-level public hospital)*, *about 8–9 years ago*. *They prescribed me several medicines after several lab tests and diagnoses*. *Now my disease is under control to some extent*. *But*, *when I stop taking medicine*, *it further comes out*.”

Indeed, the distance and the availability of healthcare facilities plays a significant role behind the care-seeking pattern. However, people visits the healthcare centres with expectations to get quality services at an affordable cost. So, we tried to seek for the answers regarding this issue which has been depicted in the next theme.

### Theme 3: Expectations from the health facilities

People usually choose a healthcare facility for their convenience or better quality service which is also cost-effective from a household perspective. The easy and accessible transportation system, amenities, quality service, availability, and accessibility of healthcare are the prerequisites of healthcare services. The majority of our respondents sought healthcare from the private clinics/hospitals because they believed that they could get at least quality healthcare services although there was a very higher treatment cost in the private clinics/hospitals. The quality of services and associated amenities were not satisfactory in sub-district level hospitals according to their observation, therefore, they often sought care at district level hospitals and even shift to private hospitals. A respondent (65) replied, “*I normally sought care from a private clinic because in nearby Modhupur Hospital (a sub-district level hospital) there were no services and scope of my treatment (e*.*g*. *diabetes)*. *Therefore*, *the authority suggested me visit a private clinic*. *However*, *that particular private clinic suggested me to admit to the Tangail Diabetic Hospital (a specialized diabetes hospital)*.” The older citizens often sought healthcare from the nearest facilities which they could reach easily with the least cost and time. Moreover, sometimes it cost more than expected if the distance of the healthcare facilities were relatively longer. One-third of the respondents preached that financial issues were the main obstacles to their treatment care. A respondent (67) stated, “*I prefer health facilities (private clinic) that are adjacent to my house*. *However*, *when I visited a nearby sub-district level hospital*, *I found nobody was there; not any nurse or even no doctor was there*. *My time and money both were wasted*. *As I have to spend my money whether I sought care from either private or public hospital*, *therefore*, *nearby private facilities are a better call for me*.” Another respondent (64) told us, “*I have a relative doctor in a private clinic who doesn’t accept any consultation fees from me*. *Moreover*, *if I have to diagnose anything*, *he manages discounts for me*. *Most of all*, *waiting time is very little in there*.”

Overall, the environment and the facilities of the health centers were not satisfactory even for the private clinics. However, some private clinics had the practice of maintaining cleanliness and better quality. Public hospitals like Upazila Health Complex (sub-district level) lagged behind in these contexts. Most of the respondents replied that the medicine ward was not clean at all and the toilets and floors were not washed daily. Moreover, there were no sufficient seats for the patients, and doctors hardly visit patients. Sometimes the caregivers and the attendants had to take shelter on the floor, and special care for older patients is unavailable.

### Theme 4: Out-of-pocket (OOP) cost—a barrier to healthcare

In Bangladesh, the high cost of medical expenses creates a significant barrier while seeking care. As a result, we wanted to highlight this burning issue and incorporated it as one of our core themes. OOP expenditure includes any payment related to medical fees, purchase of medicines (prescribed or not), user fees for care, and payments for equipment and diagnostic tests [32]. Out-of-pocket cost is generally higher for older people because morbidity complications are higher for older people compared to others. Sometimes the price of drugs becomes excessively high for them. One of them (68) replied, *“From the pharmacy*, *I came to know the price of my drugs is too high*, *therefore I have purchased only half of the listed medicine and I need to wait for further arrangement of money*.*”* In a public hospital, the treatment cost is often shared with households as public hospitals are highly subsidized in Bangladesh. However, all treatment costs are incurred by households if someone seeks care from private facilities. Some of the respondents replied that they could not seek care from a specialized doctor because of the high consultation fee which was unaffordable for them. Every time they visited the private facilities, they had to spend at least Bangladeshi Taka (BDT) 500–700 as consultation fees. Indeed, they also had to spend on other categories like medicines, diagnostic tests, hospital charges, bed fees, etc., which appeared to be a high financial burden for them. Most of the time, the older people did not go to the hospital or take any services if the cost were high. Whether it is a public or private hospital, the in-patient cost is pretty similar for both. Indeed, various surgery-related cost was higher in private facilities, which often becomes a catastrophic health burden for them. One of the respondents stated (68), “*The treatment cost is relatively higher where I usually visit for my health-related problems*. *I had to spend forty thousand BDT for only five days when I had a medical operation*.” Another respondent (66) stated, “*Sometimes I face financial catastrophes*. *Sometimes I just restrain myself from going to the doctor as my family is very needy*. *Once I was suffering from a cataract but couldn’t receive the treatment due to financial constraints*.”

OOP healthcare expense often absorbs a large proportion of the total household budget which can lead to catastrophic health expenditure (CHE). In many cases, people are forced to cut their total household consumption or, to take out loans, or to take financial help from others just to meet these health expenses which are considered as the major coping mechanisms.

### Theme 5. Coping strategies for the healthcare expenditure

Various coping strategies were observed for mitigating healthcare expenditure. During the in-person interviews, we found that the older citizens mainly met their healthcare expenditure through family funds, financial support from other family members or friends, borrowing, support from others, and even selling assets. One of them (67) replied, *“Most of the time*, *my younger son transferred money using the mobile app and then I got to purchase the medicines from pharmacies*.*”* The other respondent (69) stated, *“I received money from my elder daughter whenever I became sick*.*”* Two-thirds of the older people reported having financial difficulties during healthcare payments. Indeed, some people had to take loans and bear the debt burden for longer. Some of them worked in other people’s homes to pay the debt. Meanwhile, a few of them could not seek medical care due to financial constraints. Even if they had managed medical care, they could not buy medicines and administer them properly. Further, some older citizens solely rely on their children’s wealth. For this reason, they sometimes had to wait longer for their children’s compliance. A respondent, aged 71, stated that “*I* have huge financial problems. I have to go to Upazila Health Complexes by rickshaw. After *buying a ticket by Bangladeshi taka 5*, *I have to take medicine from outside the hospital*. For mitigating treatment costs, I had to borrow three lac Bangladeshi taka from the bank. Now I am repaying the money by working in other’s houses which is difficult for me.”

This study tried to capture important suggestions from older citizens regarding healthcare management and treatment care in public hospitals. However, two-thirds of the respondents mentioned various issues related to the hospital management and other support systems; such as the need for separate and furnished waiting rooms where they could easily sit down, ensuring availability of health personnel including physicians, nurses for older care, 24/7 hospital services, robust referral systems, quality healthcare services, older-friendly accessibilities. They also strongly advised on improving the cleanliness of the facilities and ensuring the availability of medical equipment for investigations in public hospitals as they can’t afford many diagnostic tests in private sectors. One of the respondents, aged 68, stated, “*In hospitals*, *it would be better if we get healthcare services in the separate chamber/ segments where only older patient would be treated; such as establishing an older friendly health unit where an older citizen would go and get treatment faster than other age groups*. *Currently*, *many hospitals have insufficient doctors*, *and nurses than needed*, *so the number of doctors and nurses has to be increased and those who are already recruited have to do their duty properly*. *Doctor visit fees should be further reduced*, *and the government should be strict about it*. *The price of the drug should be written on the packet*. *Poor people should be given a card with which they can get a 50% discount on drugs and free medicines from the government hospital*. *People are not getting exactly what the government is providing; they need to be monitored*.”

## Discussion

Improving health and well-being is a global priority in the latest sustainable development goals (SDGs); SDG-3 focuses exclusively on ensuring healthy lives and promoting well-being for all, regardless of age. As the share of the aged population has been growing rapidly and they usually suffer from various chronic diseases, it is crucial to identify their current experiences in seeking health care so that appropriate strategies could be designed to improve their health and well-being. This study tried to capture the individuals’ experiences regarding healthcare-seeking and accessibility, affordability, and coping strategy for older citizens in Bangladesh.

Health status is often measured through the presence of any disabilities that limit full participation in activities. The overall health status of older citizens is found to be poor in Bangladesh. It might be seen that all of the older citizens suffer from various illnesses and disabilities, such as hypertension, gastric ulcer, body aches, diabetes, flu/cough etc. Like other settings, Bangladesh has been experiencing a growing burden of NCDs and 66% of the older people suffers from various NCDs [33]. Several studies have investigated regarding this issues in near-by countries. For example, NCDs contributed to 70% of mortality and morbidity among the population in Bhutan, 50% of the total health burden in terms of mortality in Nepal, 75% of total deaths, and 62% of mortality in India [34]. Due to rapid urbanization, unhealthy diet, increased life expectancy, and lifestyle changes, the rate of cardiovascular diseases, including hypertension, has increased over the years [35]. The prevalence of hypertension in Bangladesh has increased from 17.9% in 2010 to 21% in 2018 [36,37]. Diabetes, the other overwhelming disease, is significantly higher in older than in young people that we have found in our study [38]. People over 65 years are more likely to get affected by gastric ulcers or gastrointestinal disorders, and more than 80% of the death caused by gastrointestinal disorders are happened to be the people of this age group [39]. Moreover, eye infections and body ache called musculoskeletal problems are also widespread for older people in Bangladesh. Besides this, we observed the prevalence of comorbidities is high among older people as found in an earlier study and they require more specialized care [40]. Indeed, the public investment is still focused on maternal and child health issues [41,42]. Therefore, improvements to older-friendly health infrastructure are necessary to improve the health of older citizen.

We observed that the distance of healthcare facilities and lack of an older-friendly transportation system was the vital issues whicle seeking care. Due to physical limitations, older people often faced difficulties for choosing to their desired health facilities; as the communication barriers still exist in Bangladesh [20]. Our study revealed that, overall, hospitals’ cleanliness and surrounding environment is not satisfactory for them. Although few private facilities now maintain cleanliness and relatively better quality, the primary and secondary public hospitals like Upazila Health Complex (sub-district level hospital) lagged in this context, and thus older people are often unwilling to go to such public hospitals that we found during a discussion with respondents [43]. We observed that most of the participants preferred private healthcare facilities, although such care is unaffordable for poor and marginalized people in Bangladesh [43]. The current study also observed that some of the respondents did not seek health care due to high perceived out of pocket costs. There is evidence from developing countries including Bangladesh that due to financial barriers older people usually do not seek care [29]. In this regard, the government should take the initiative to devise special free out-door service units for providing quality care in public hospitals and special free transport services for the older citizen. Health care facilities should focus on improving the quality of health services and strengthening the health care delivery system with referral networks as the existing services are quietly insufficient.

There is some evidence that households often spend more on older care due to various disease complexities and extra care. A study conducted in neighborhood of India found that households’ older member’s monthly per capita healthcare expenditure is 3.8 times higher than younger ones, which is catastrophic for many households [44]. From this study, it seems that older citizens often face financial crises for seeking care which hurts the mental health of older adults [45]. This is also true for Bangladeshi older people as OOP cost bears one of the lion-share for healthcare financing in Bangladesh which is increasing alarmingly. The current study also observed that the older people use different coping strategies like family funds, support from relatives, borrowing and selling assets for their health care expenditure. A study in rural Bangladesh also identified similar coping strategies [29]. Universal social security programs such as universal old-age pension, and old age allowance could be initiated so that they could be free from anxieties and depressions in later life. A similar program was already initiated in many neighborhood countries such as old-age allowance in Thailand, social pensions in Vietnam, senior citizens’ allowance in Nepal etc [46]. The Government of Bangladesh has several Social Safety Net (SSN) Programmes from which older people get some direct and indirect benefits. The government took few initiatives for the older people, such as the pension system, retirement benefits and other initiatives under various programmes. The ‘Old Age Allowance’ policy was introduced for the poor older population of the nation. Still, the coverage is not yet entirely satisfactory as a large number of people from the older population remains outside the ambit of these programmes. Ministry of Health and Family Welfare of Bangladesh still has room for developing new strategies and revising existing policies to address this older age group. Indeed, social health insurance can therefore be viewed as an essential component of financial protection as it aims to make healthcare affordable and accessible to all older citizens.

This research has several limitations. Since the participation was voluntary, a certain bias could not be completely avoided. Further, the findings cannot be generalized to other populations in Bangladesh as a nationwide survey should be required for such interpretations. Nevertheless, given the focused nature of guidelines and the consistency of the data, this study did provide vital information about older healthcare. Again, recall bias might be associated, considering the care-seeking pattern, cost of treatment and even the reporting of specific diseases. Recall bias and incorrect reporting may be underestimating or overestimating the actual situation. All of the above represents possible avenues for further research on this topic.

### Conclusion

With the increase in older people, the health burden is also on the rise, but still there is inadequate data on health care seeking experiences of older population in Bangladesh. There must be strategic implementation of policies focusing on the barriers and shortfalls, which prevent them from seeking health care. This study has documented the experiences of older people regarding healthcare-seeking and accessibility, affordability, and healthcare coping strategy. The study findings suggest that the improvement to older-friendly health infrastructure is necessary and universal pension schemes and social health protection schemes should be introduced to improve the health of older citizens in Bangladesh.

## Supporting information

S1 Text Guideline for in-depth interview (IDI). (DOCX)
