# Supplementary material for: Healthcare-seeking experiences of older citizens in Bangladesh: A qualitative study
Source: PLOS Glob Public Health. 2023 Feb 8;3(2):e0001185. doi: 10.1371/journal.pgph.0001185 (PMC10022267; doi:10.1371/journal.pgph.0001185)
Supplement: S1 Text — (DOCX) [file pgph.0001185.s001.docx]

**S1 Text**

Guideline for In-depth Interview (IDI)

Questionnaire ID:

Name and short address:

**Information about the respondent:**

- The current age of the elderly person
- Educational Qualification
- Occupation
- Details information regarding the family (total family member, monthly income, monthly expenditure, etc.)

**Current physical state:**

- What is the current physical health state?
- Are you suffering from any diseases right now? (Names of the diseases, if any, types of problems faced due to that illness, etc.)
- Are you suffering from any mental illnesses in the absence of physical disease?
- Were you suffering from any other diseases before your current illness? (Names of the conditions before the present illness, illness period, types of problems faced due to those illnesses, etc.)

**Treatment information:**

- Do you seek treatment or visit the health facilities during illness? (If you don’t seek treatment, what are the reasons behind that etc.)
- Do you face any difficulties/obstacles when seeking treatment? (If yes, what are the types of difficulties faced? E.g., financial, physical, mental, etc.)
- Did you seek any treatment for your previous illness? (If not, what were the reasons?)
- Where do you usually go for the treatment? (Ask in detail in case of multiple treatment facilities)
- Why do you go/prefer that treatment facility? (Ask in detail about traveling time, waiting time, etc.)
- Do you seek treatment from any other place besides the facility mentioned above? (If yes, ask in detail about traveling time, waiting time, etc.)
- Did you get any help or advantages because of your age from the hospital staff or other patients? (For example, doctor chamber visits ahead of appointment time)
- How is the quality of care at your preferred treatment facility? (Effectiveness of the treatment, types of medicines prescribed, the need for repetitive doctor visits, etc.)
- Are there any attendants at your home or the healthcare facility to take care of you? (Availability of attendant/s to accompany you to the hospital, to look after you at home, etc.)
- How is the environment of your preferred treatment facility? (Cleanliness, toilet facilities, etc.)
- What other facilities would make your experience more convenient in the healthcare facility?

**Healthcare expenditure information:**

- How much is the healthcare expenditure at your preferred treatment facility? (Consultation fees, medicines, diagnostic fees, etc.)
- How do you pay for the treatment cost? (Who pays for it, the need to take a loan, the need to cut down food expenses, etc.)
- What steps from the government should be taken to lessen your burden of healthcare expenditure?
- Have you ever been deprived of medical treatment due to a lack of money? (If yes, ask in detail)

**Other information:**

- Are you a member of any social safety program, e.g., elderly pension, allowances, etc.? (If yes, ask in detail about the name of the program and the type of benefits received)
- Are you a member of the social safety program SSK? (If yes, ask in detail about how they became a member, whether they have the knowledge of how to use the card or not, benefits received after becoming a member, etc.)
